# Liposomal 5-Fluorouracil Polymer Complexes Facilitate Tumor-Specific Delivery: Pharmaco-Distribution Kinetics Using Microdialysis

**DOI:** 10.3390/pharmaceutics14020221

**Published:** 2022-01-18

**Authors:** Wen Wang, Paul Joyce, Kristen Bremmell, Robert Milne, Clive A. Prestidge

**Affiliations:** UniSA Clinical & Health Sciences, University of South Australia, Adelaide, SA 5000, Australia; wen.wang@mymail.unisa.edu.au (W.W.); paul.joyce@unisa.edu.au (P.J.); Kristen.bremmell@unisa.edu.au (K.B.)

**Keywords:** 5-fluorouracil, liposomes, cancer nanomedicine, tumor targeting, anti-cancer, drug delivery

## Abstract

Liposomes are widely used as carriers for anticancer drugs due to their ability to prolong the retention of encapsulated drugs in blood plasma while directing their distribution increasingly into tumor tissue. We report on the development of stealth liposomal formulations for the common chemotherapy drug 5-fluorouracil, where pharmacokinetic studies were undertaken using a microdialysis probe to specifically quantify drug accumulation in tumor, which was contrasted to drug exposure to healthy tissue. Greater accumulation of the drug into the tumor than into healthy subcutaneous tissue was observed for neutral and cationic liposomal 5-fluorouracil polymer complexes in comparison to the conventional delivery by an injected solution. Increased drug accumulation in tumor also correlated to reduced tumor growth. This research has generated new mechanistic insight into liposomal-specific delivery to tumors with potential to improve the efficacy and reduce the toxicity of chemotherapy.

## 1. Introduction

Liposomes are widely recognized as effective carriers for anticancer drugs due to their high biocompatibility and their capacity to prolong retention of the encapsulated drugs in plasma and increase their distribution into tumors [1,2,3]. The functionality of liposomal formulations can be modulated through controlling the lipid composition and by employing a range of surface-engineering strategies [4], such as the inclusion of polyethylene glycol (PEG) lipids with stealth-like characteristics or targeting ligands that afford site-specific localization. For example, cationic liposomes can target angiogenic endothelial cells in tumors selectively [5,6,7], due to key biological characteristics of angiogenic tumor blood vessels [8]. Tumor endothelia lack the glycocalyx layer that usually covers vascular endothelial cells of normal healthy tissue, thus exposing a negatively charged cell surface. Among the microvessel-associated target structures, there are negatively charged cell surface molecules such as glycoproteins, anionic phospholipids, and proteoglycans [9,10,11], which exist as potential binding sites for cationic liposomes and offer the possibility for selectively targeting of diagnostic or therapeutic agents contained within cationic liposomes to tumor endothelial cells.

The most-studied anticancer drug to have successfully been encapsulated in cationic liposomes for passive tumor-targeting is paclitaxel (EndoTAG-1^®^). EndoTAG-1 is composed of the established cytostatic drug paclitaxel combined with neutral and positively charged lipids, which include DOTAP and DOPC, and has been shown to significantly retard tumor growth and delay the incidence of metastatic disease. Detailed analysis revealed that this formulation resulted in a mechanistic switch from direct toxicity to tumor cells toward effects on the tumor vasculature [12]. Importantly, in a Phase II trial, treatment with cationic liposomal paclitaxel plus gemcitabine showed beneficial efficacy against advanced pancreatic cancer and increased survival [13].

Despite cationic liposomal formulations for anticancer agents exerting positive outcomes for treating cancer in preclinical and Phase I and II studies [13,14], little research has investigated how liposomes alter the distribution of encapsulated chemotherapeutic agents within tumor tissue. In vivo imaging has shown that PEG-coated cationic liposomes are retained in tumor tissue for a longer period than PEG-coated neutral liposomes [15]. This study also reported that the disposition of the anticancer agent, oxaliplatin, in tumor tissues when administered intravenously in cationic PEG liposomes was different from that of neutral liposomes; namely, that encapsulated anticancer drugs may distribute into tumor tissue more rapidly from cationic liposomes than from neutral liposomes and may also be eliminated more slowly from tumor tissue [15]. The authors measured oaxaliplain (as Pt) in alkaline digests of tumors and observed maximum intratumor accumulation of oxaliplatin 6 h post-injection of a solution, which gradually decreased to near zero levels by 48 h. A three-fold higher maximal concentration of oxaliplatin was achieved with PEG-coated neutral liposomes, but this value was reached only after 24 h, and at 48 h the level was less but still higher than the maximal level obtained with a solution of the drug. Meanwhile, with PEG-coated cationic liposomes an almost three-fold higher level of drug was obtained after 6 h than with PEG-coated neutral liposomes while concentrations continued to increase up to 48 h post-injection.

Considering this, an experimental approach that affords dynamic, time-dependent pharmacokinetic analysis of liposomal anti-cancer drugs within plasma, healthy subcutaneous (*s.c.*) and tumor tissue is therefore desired to optimize liposome composition for tumor targeting. Microdialysis probe methods enable quantification of drug concentrations within the extracellular fluid (ECF) of tumors, with studies demonstrating its application in both xenograft animal models and in patients with accessible tumors [16,17,18,19,20]. By obtaining continuous drug concentration profiles a comparison of exposure in the ECF of the tumor and plasma within the same animal can be obtained [21,22], which importantly avoids the need for excessive sacrificing of animals for the collection of tissue samples. However, the greatest advantage of microdialysis is the ability to quantify free drug within tumor tissue, since only chemotherapeutic drug reaching tumor sites can provide therapeutic effects, while drug delivered to normal tissues has the potential to generate toxicity. Thus, drug delivery to the tumor is determined not only by concentrations in plasma but also by its distribution from the plasma into the extracellular fluid of the tumor [23,24]. Some tumors fail to respond even on exposure to apparently cytotoxic concentrations in plasma. At best, systemic exposure in plasma serves only as a surrogate measure of exposure to drug in the tumor. Thus, exposure to anticancer drugs in the ECF of tumor cells is considered to serve as a better representation of efficacy than drug exposure in plasma.

The main aim of the present study was to assess the impact of the different liposomal formulations (including neutral and cationic PEGylated liposomes) on the exposure (i.e., area-under-the-curve, AUC) of 5-fluorouracil (5-FU) to tumor tissue relative to healthy tissue. Since 5-FU is a highly permeable compound, polymer complexes were formed through electrostatic interactions with ternary copper and polyethyleneimine (PEI) to retard premature drug release from the liposomal formulations and thus, enhance tumor exposure to the drug [25]. Microdialysis technology was employed to evaluate the uptake and disposition of 5-FU polymer complexes after being encapsulated in both neutral and cationic liposomes. The availability and time course of 5-FU in the tumor and adjacent healthy *s.c.* tissue was compared after simultaneously measuring the free concentrations of 5-FU in the interstitial fluid of both tissues. To the best of our knowledge, the present study represents the first attempt at utilizing microdialysis to evaluate the uptake and disposition of an anti-cancer drug encapsulated in both neutral and cationic liposomes into tumor tissue.

## 2. Materials and Methods

### 2.1. Materials

1,2-distearoyl-*sn*-glycero-3-phosphatidylocholine (DSPC), 1,2-dimyristoyl-*sn*-glycero-3-phosphoethanolamine-*N*-(methoxy(polyethylene glycol)-2000) (DSPE-mPEG2000), and cholesterol were purchased from Avanti Polar Lipids, Inc. (Alabaster, AL, USA). O,O’-ditetradecanoyl-*N*-(α-trimethyl ammonio acetyl) diethanolamine chloride (DC-6-14) was obtained from Sogo Pharmaceutical (Tokyo, Japan). AnalaR-grade sodium chloride, potassium chloride, calcium chloride dehydrate, and sodium bicarbonate were purchased from BDH Chemicals (Poole, England). Water was purified using a Milli-RQ Ultrapure Water System (MerckMillipore, Burlington, Massachusetts, USA). Polyethyleneimine (MW 800), copper acetate, chloroform, 5-fluorouracil (5-FU), and 5-chlorouracil (5-CU) were obtained from Sigma (St. Louis, MO, USA). The safe handling of 5-FU followed Standard Operating Procedures approved by the University of South Australia.

### 2.2. Synthesis of 5-FU Complexes with Copper Crosslinked Polyethyleneimine

Copper-crosslinked polyethyleneimine Cu-PEI was prepared by adding molar equivalents of polyethyleneimine into solution of copper acetate in water (0.1 M, 1 mL) with continuous stirring until a homogenous purple blue solution was obtained. The resulting solution was heated to 60 °C for 10 min before incubation with a 5-FU solution (8 mg/mL; based on 5-FU solubility in PBS) for 10 min with a final molar ratio of Cu:PEI:5-FU at 1:1:1. The resultant 5-FU-Cu-PEI complex was centrifuged (5000× *g*, 10 min) and washed to remove any free 5-FU.

### 2.3. Fabrication & Characterization of Liposomal 5-FU Formulations

Three liposomal 5-FU formulations were synthesized in the current study, being (1) neutral liposomes with 5-FU (5-FU LiPo), (2) neutral liposomes with 5-FU-Cu-PEI (5-FU-Cu-PEI LiPo), and (3) cationic liposomes with 5-FU-Cu-PEI (5-FU-CuPEI LiPo+). Liposomes were prepared using the conventional thin-film hydration method originally developed by Bangham and colleagues [26] with some modifications. Liposomes were prepared by dissolving phospholipids and cholesterol in chloroform to give a final lipid concentration of approximately 10 mM. The lipid composition used throughout this study was selected based on the optimal encapsulation efficiency within neutral liposomes with varying cholesterol and DSPE-PEG2000 concentrations. That is, cholesterol concentrations were varied between 30 and 50 mol% and DSPE-PEG2000 concentrations were varied between 2 and 10 mol%. The lipid solutions were transferred to a round-bottom flask for evaporation using a rotary evaporator (R-210 Rotavapor, BUCHI, Switzerland) under vacuum (V-700 Vacuum Pump, BUCHI, Switzerland) at room temperature for 2 h to produce a thin lipid film at the wall of the round bottom flask. The lipid film was subsequently rehydrated with either 5-FU or 5-FU-Cu-PEI, equivalent to a 5-FU concentration of 8 mg/mL (based on 5-FU solubility in PBS), in phosphate buffer (pH 7.4) using the same rotary evaporator for another 2 h at 65 °C (B-491 Heating Bath, BUCHI, Switzerland). The multi-lamellar vesicles (MLV) were obtained directly after hydration, sonicated for 1 min, and extruded through membranes for 15 cycles to obtain the desired size range (<200 nm). The liposomal suspension was ultracentrifuged (Beckman Coulter ultracentrifuge, type 70.1 Ti Rotor, 12 × 13.5 mL) at 61,000× *g* at 4 °C, and the concentration of 5-FU in the known volume of supernatant measured by HPLC (Section 2.5.3). The quantity encapsulated was estimated from the difference between the known amount in buffer and the amount in the supernatant and encapsulation efficiencies were calculated based on the initial amount of 5-FU added to the hydrated lipid. Equation (1) was used to calculate encapsulation efficiency (EE%), as provided below:

(1) $$\mathrm{EE}\%=\left[1\;-\frac{M_{f}}{M_{t}}\right]\;\times \;100\%$$

where M_f_ is the amount of free drug remaining in solution and M_t_ is the total amount of drug added to the lipid mixture. 5-FU loading was calculated based on the mass of drug encapsulated versus the mass of lipid within the liposomes.

The particle sizes and zeta potential of the liposomes were measured using a Malvern Zetasizer Nano-series (Malvern Instruments Ltd., Malvern, UK).

### 2.4. In Vitro 5-FU Release Studies

The release of encapsulated 5-FU was examined by dialysis against phosphate buffer saline (PBS; 50 mM, pH 7.4). Briefly, liposomal formulations equivalent to 1 mg 5-FU were placed into dialysis tubing (MWCO 12000–14000 Da, Fisher Scientific Inc., Hampton, NH, USA) with both ends and the bag suspended in PBS (10 mL, 37 ± 0.5 °C) with stirring (100 rpm). At predetermined time points, aliquots (100 µL) were taken and replaced with the same amount of fresh buffer [27]. Concentrations of 5-FU were quantified using HPLC, and all experiments conducted in triplicate. Adjustment was made for the replacement volume when the concentrations were converted to the amount of 5-FU released.

### 2.5. In Vivo Pharmacokinetic Study in Tumor-Bearing Sprague-Dawley Rats

#### 2.5.1. In Vivo Tumor Model

All rats used in this study were obtained from SA Pathology (Adelaide, Australia). They were housed under pathogen-free conditions at room temperature (22 °C) with a circadian light rhythm of 12 h and free access to food and water. The procedures were approved by the SA Pathology Animal Ethics Committee. W256 cells (Tohoku) were used in this study as a model cancer cell-line and were harvested and the percentage of viable cells determined using 1% trypan blue solution in a Neubauer chamber. The cells were centrifuged (ROTINA 48, Hettich Zentrifugen, Tuttlingen, Germany) at 310 g and resuspended in a 50 mL centrifuge tube with 1 mL of PBS. The cells were dispersed and stored under ice pending transplantation into the rats. Sprague-Dawley rats (80 to 100 g) were anaesthetized and a suspension containing approximately 10^7^ W256 cells (Tohoku) in 1 mL PBS was injected into the right flank. The rats were returned to their cages and monitored until they woke up. The tumor diameter was determined daily using a caliper. Tumor volume was calculated as 0.5 length (mm) × width (mm)^2^. The experiments were performed when the tumor volume reached 2–3 mL.

#### 2.5.2. Microdialysis Setup

Rats were anesthetized with isoflurane inhalation (Attane, Pharmtech, Australia) and a cannula (PE50 tubing with silicone tip, 0.51 mm × 0.94 mm) was inserted into the right jugular vein for the collection of blood samples. A guide split tubing with needle was inserted into tumor tissue (right flank) and healthy subcutaneous (*s.c.)* tissue at a corresponding site on the left flank through separate small incisions of the skin. The needle was removed and CMA/20 probes (CMA/Microdialysis, Sweden; membrane OD: 0.5 mm; shaft OD: 0.67 mm) were inserted through the split tubing and secured to the surrounding skin. Finally, the guide tubing was split away. The probes were perfused with perfusion media composed of sodium chloride (6.76 g/L), potassium chloride (0.0900 g/L), calcium chloride dehydrate (0.117 g/L), and sodium bicarbonate (0.225 g/L) in MilliQ, adjusted to pH to 7.4 with hydrogen chloride (based on previous studies [28,29]). Perfusion medium was pumped from a 1 mL BD syringe (Becton Dickinson, Macquarie Park, NSW, Australia), through the inlet tubing of the microdiaysis probe at the rate of 2 μL/min using a syringe pump (Razel A-99, Extech Equipment Pty. Ltd., Boronia, VIC, Australia). Dialysate was collected via the outlet tubing into plastic micro-vials held in a CMA microfraction collector.

#### 2.5.3. 5-FU Pharmacokinetics from Liposomal Formulations

Following a 30 min period for establishing equilibrium between perfusing medium and extracellular fluid (ECF) of the tissue, the rats were administered conventional or liposomal 5-FU through the tail vein (10 mg/kg, 0.4–0.7 mL/animal). Three types of 5-FU liposomal formulations were included: (1) 5-FU LiPo, (2) 5-FU-PEI-Cu LiPo, and (3) 5-FU-PEI-Cu LiPo+. After reconstituting the liposomal pellet with an equal volume of phosphate buffer, the appropriate volume was administered *i.v.* into the tail vein to ensure a dose of 10 mg/kg of 5-FU. Samples of blood and approximately 200 µL of microperfusate (i.e. perfusion fluid collected from microdialysis) were collected at selected times by a microfraction collector (CMA 142, CMA/Microdialysis, Sweden) every 10 min after injection. The blood was centrifuged at 1485× *g* for 10 min (Eppendorf centrifuge). The supernatant (plasma, 100 µL), along with dialysate, were stored at −20 °C pending analysis. Rats remained anesthetized prior to sample collection and were monitored carefully throughout the experiment. At the end of the study, they were humanely killed by cervical dislocation under anesthesia.

#### 2.5.4. Recovery of 5-FU Determination

Recovery of 5-FU was estimated from loss of the drug in vivo by reverse dialysis. Following drug administration and completion of sampling, the blank perfusion medium was replaced with a medium containing 5-FU (2 µg/mL) and, after an equilibration period of 30 min, samples of dialysate were collected every 10 min for at least 1 h. The in vivo recovery was calculated using Equation (2):

(2) $$\;\mathrm{In}\;\mathrm{vivo}\;\mathrm{recovery}=\left[\frac{C_{\mathrm{in}}\;{-\;C}_{\mathrm{out}}}{C_{\mathrm{in}}}\right]\times \;100\%$$

where C_in_ and C_out_ are the concentrations of 5-FU in the perfusion medium and dialysate, respectively.

#### 2.5.5. 5-FU Concentration Determination using High-Performance Liquid Chromatography

The concentrations of 5-FU in all the above samples were determined by HPLC with UV-detection, within two weeks of their collection. The HPLC method used for this study was based on a method validated in a previous study conducted in our laboratory [30]. Briefly, plasma (100 µL) with 20 µL internal standard (5-CU, 5 µg/mL in MilliQ water) was extracted with ethyl acetate (1 mL) by shaking with a vortex-mixer and centrifuging for 10 min at 1485× *g*. Supernatant (900 µL) was transferred to a clean Eppendorff tube and evaporated under a stream of nitrogen at room temperature. The residue was reconstituted with 100 µL milli-Q water. Perfusate (20 µL) with 20 µL internal standard was diluted with 40 µL Milli-Q water. Forty µL of the reconstituted extract from plasma or the diluted perfusate was injected onto the HPLC column.

HPLC was performed using a LC-20AD pump, a SIL-20A auto autoinjector, a PDA detector, and a DGU-20A_3_ system-controller (Shimadzu, Kyoto, Japan). The analytes were separated on a Grace C_8_ column preceded by a guard column (4 × 3 mm, C_18_, Phenomenex), compressed in a guard cartridge holder (Phenomenex, CA, USA), and operated at room temperature (22 °C). The mobile phase (Milli-RQ water, sonicated for 5 min before use) was pumped at a flow rate of 0.8 mL/min. Adequate chromatographic separation of 5-FU and 5-CU was achieved. Retention times of 5-FU and 5-CU were ~6.91 and ~10.2 min, respectively, for both perfusate and plasma samples. Calibration curves were linear over the range from 0.02 to 0.50 ug/mL (low end, r^2^ = 0.997) and 0.50 to 100 ug/mL (high end, r^2^ = 0.999) in plasma and from 0.01 to 0.50 ug/mL (low end, r^2^ = 0.999) and 0.50 to 50.0 ug/mL (high end, r^2^ = 0.999) in perfusate. The lower limits of quantification for 5-FU in plasma and perfusate were 0.02 μg/mL and 0.01 μg/mL, respectively, defined by the lowest calibration point within 20% deviation from the nominal concentration and coefficient of variation (%CV) of replicate injections (*n* = 6) less than 20%. The accuracy of the quality control samples was within 11.8% for plasma (0.05, 0.5, 4, and 20 µg/mL, *n* = 7 for each concentration) and 13.0% for perfusate (0.03, 0.5, 1, and 10 µg/mL, *n* = 7 for each concentration) while the precision (%CV) of the quality control samples was within 11.1% for plasma and 12.8% for perfusate, respectively.

### 2.6. In Vivo Tumour Efficacy Study

Five groups (*n* = 6) of tumor-bearing Sprague-Dawley rats were used for assessing efficacy of each formulation on the growth of the tumor. The same formulations as for the pharmacokinetic study (Section 2.5) were used, but they were administered as repeated doses for five consecutive days (all at doses equivalent to 10 mg/kg of 5-FU). The key parameter used for the assessment of efficacy was the tumor size. Using Thomas and colleagues’ [25] work as a guidance where the variation of the tumor size was ~30% of the mean, setting a difference of 40%, significance as 0.05, and power as 0.8, six animals were used in each experimental group to warrant statistically meaningful results. The sizes of the tumors were measured using a caliper. Tumor volume was calculated as 0.5 length (mm) × width (mm)^2^. Treatments were initiated five days after injection of the suspension of tumor cells into rats. For minimum distress to the animals, the experiment was terminated on day 12 and the animal humanely killed once the diameter of the tumor had reached 4 cm.

### 2.7. Statistical Analysis

The concentrations of 5-FU in ECF represented time-averaged concentrations over the dialysate collection interval and were corrected for in vivo recovery. Non-compartmental analysis of the data for concentrations of 5-FU in plasma, *s.c.* tissue and tumor tissue versus time was performed using Phoenix WinNonlin 6.4 (Pharsight, Mountain View, CA, USA), without weighting. The minimum number of data points that WinNonlin used to make the calculation of the terminal rate constant was four for both plasma and tissue data. AUC (representing AUC to infinity in plasma and in dialysate) was calculated using the linear trapezoid method with extrapolation beyond the last measured concentration using the terminal rate constant. A one-way ANOVA was performed with IBM SPSS Statistics 21 (IBM, Armonk, New York, NY, USA). A homogeneity-of-variance test was carried out to determine whether an assumption of equal variances was valid. A Tukey’s post-hoc test was performed when equal variances was observed while a Dunnett’s T3 test was performed when variances were not equal. The level of significance was set at *p* < 0.05.

## 3. Results

### 3.1. Optimizing 5-FU Encapsulation Efficiency in Liposomes

**Figure 1:**
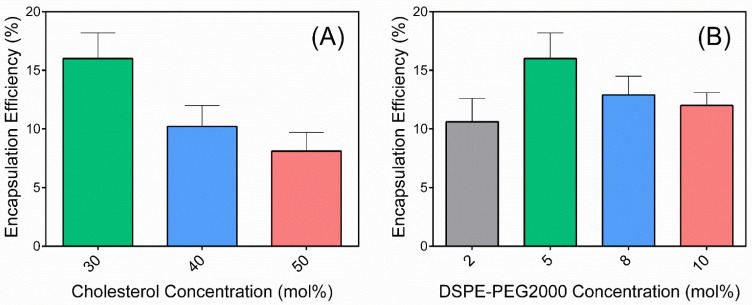
Optimization of 5-FU encapsulation efficiency through varying (**A**) cholesterol concentration (using a DSPE-PEG2000 concentration of 5 mol%) and (**B**) DSPE-PEG2000 concentration (using a cholesterol concentration of 30 mol%). Data represent mean ± S.D. (*n* = 3).

The lipid composition of liposomes used within this study was selected based on the optimal encapsulation efficiency of 5-FU. Specifically, the molar ratio of cholesterol and PEG phospholipids (i.e., DSPE-PEG2000) was varied and the subsequent encapsulation efficiency was determined. Increasing cholesterol content from 30 to 50 mol% within liposomes negatively impacted encapsulation efficiency, while the optimal DSPE-PEG2000 concentration was observed to be 5 mol% (Figure 1). Subsequently, liposomes were fabricated with 30 mol% cholesterol and 5 mol% DSPE-PEG2000 for the remainder of the study, which led to mono-dispersed liposomes between 114 and 128 nm in diameter (PDI 0.168) with a 5-FU encapsulation efficiency of 16.0 ± 2.2%, which corresponded to a drug loading (i.e., mass of encapsulated drug versus mass of lipid) of 16.6 ± 2.3%. The zeta potential was modulated through changes in lipid composition, with liposomes prepared with DSPC/CHOL/DSPE-PEG2000 at a molar ratio of 65:30:5 mol% (LiPo) exerting a zeta potential of −19.6 ± 1.6 mV compared to +9.2 ± 0.8 mV for liposomes with a lipid composition of DSPC/DC-6-14/CHOL/DSPE-PEG2000 at a molar ratio of 30:35:30:5 mol% (+LiPo).

### 3.2. In Vitro 5-FU Release from Liposomal Formulations

**Figure 2:**
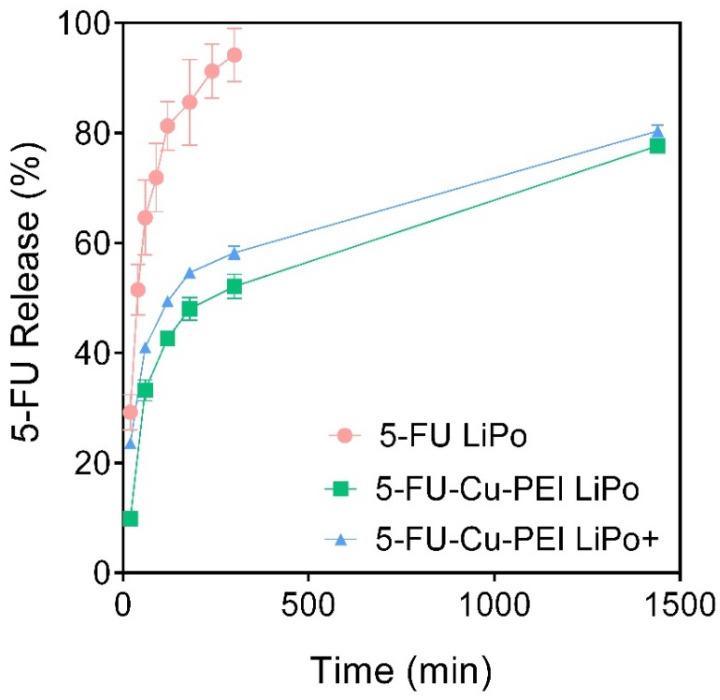
In vitro 5-FU release from 5-FU LiPo (red circles), 5-FU-Cu-PEI LiPo (green squares), and 5-FU-Cu-PEI LiPo+ at pH 7.4, 37 °C. Data represent mean ± S.D. (*n* = 3).

The in vitro release of encapsulated and uncomplexed 5-FU from liposomes (5-FU LiPo) was rapid with >50% of the payload being expelled from the liposomes after just 40 min (Figure 2). In contrast, complexing 5-FU with Cu-PEI created a larger molecule that was not as permeable as non-complexed 5-FU and therefore the in vitro drug release kinetics was significantly reduced, with approximately half released in 400 min and 75% in 24 h for both neutral and cationic liposomes. The lipid composition did not significantly alter in vitro release kinetics of complexed 5-FU.

### 3.3. Pharmacokinetics of Liposomal 5-FU Formulations

**Figure 3:**
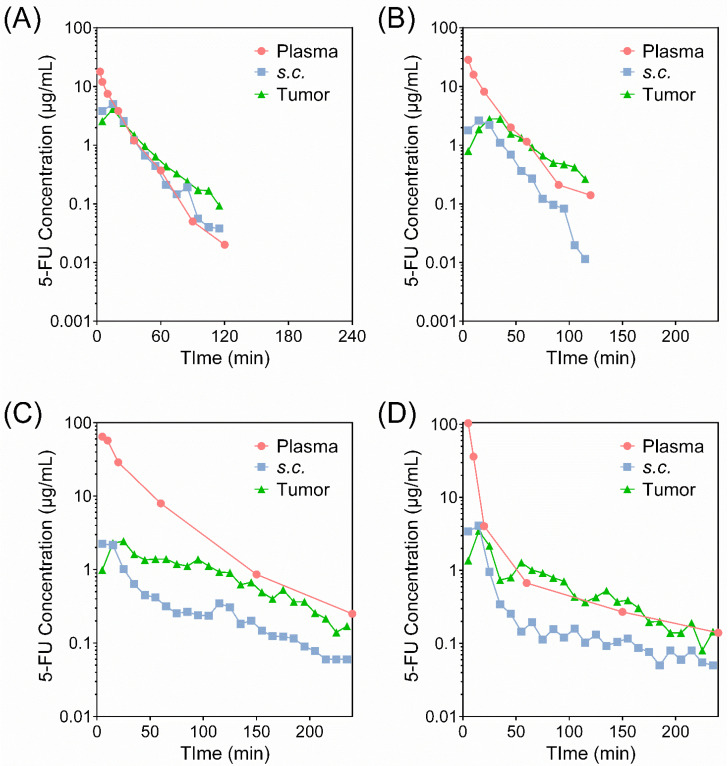
Concentrations of 5-FU versus time in plasma and the ECF of healthy *s.c.* tissue and tumor tissue following i.v. administration (10 mg/kg) of (**A**) 5-FU solution, (**B**) 5-FU LiPo, (**C**) 5-FU-Cu-PEI LiPo, and (**D**) 5-FU-Cu-PEI LiPo+. Mean values are plotted on logarithmic–linear coordinates (*n* ≥ 5).

Figure 3 shows the concentration of 5-FU in plasma and the extracellular fluid (ECF) of subcutaneous (*s.c.*) and tumor tissue over time after intravenous (*i.v.*) administration of various 5-FU formulations to tumor-bearing rats at a dose of 10 mg/kg. Corresponding pharmacokinetic parameters are included within Table 1. N.B. 5-FU polymer complexes were only investigated in liposomal systems, and not as a free solution, due to the inability for the large polymer complex to cross cellular membranes, and thus exert anti-cancer efficacies.

**Figure 4:**
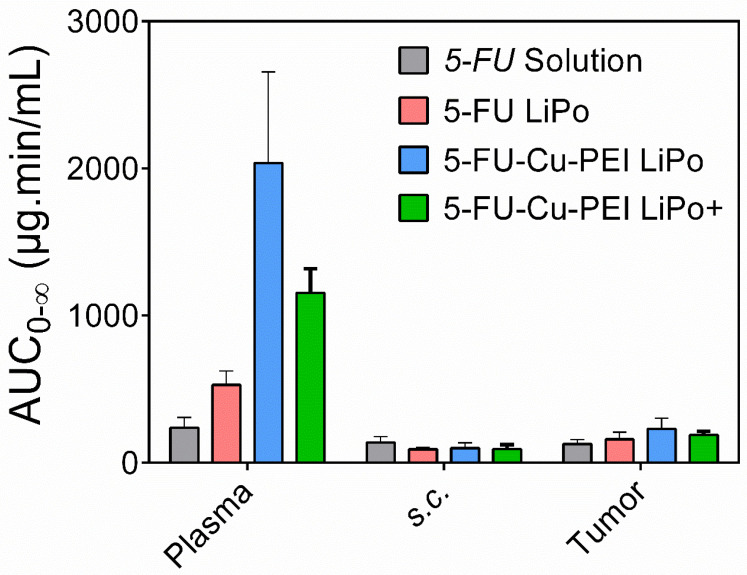
AUC of 5-FU in plasma and the ECF of healthy *s.c.* tissue and tumor tissue following i.v. administration of various 5-FU formulations (10 mg/kg). Data represent mean ± S.D. (*n* ≥ 5).

Inclusion of 5-FU within liposomes prolonged drug exposure within plasma and tumor tissue, irrespective of the lipid composition or drug complexation, when compared to the 5-FU solution. Importantly, this highlights the capacity for liposomes to reduce clearance kinetics of 5-FU. The pharmacokinetic profiles of complexed 5-FU in both neutral and cationic liposomes revealed that complexation is a promising approach for further prolonging exposure within plasma and tumor tissue, while reducing the exposure of healthy *s.c.* tissue compared to the pure drug and uncomplexed 5-Fu in liposomes. This is highlighted in Figure 4, which contrasts the area-under-the-curve (AUC_0–∞_) for 5-FU in plasma, *s.c.,* and tumor tissue. In the case of both 5-FU-Cu-PEI LiPo and 5-FU-Cu-PEI LiPo+, the mean residence time (MRT) in *s.c.* tissue increased to 69.9 ± 8.5 min (*p* < 0.001) and 39.3 ± 14.9 min, respectively, (compared to 26.3 ± 9.2 min for 5-FU solution), as well as in tumor tissue to 99.5 ± 24.8 min (*p* < 0.001) and 96.4 ± 27.0 min, respectively, (compared to 36.1 ± 10.3 min for 5-FU solution) (*p* < 0.01), thus highlighting the sustained exposure of complexed drug within each liposome.

**Figure 5:**
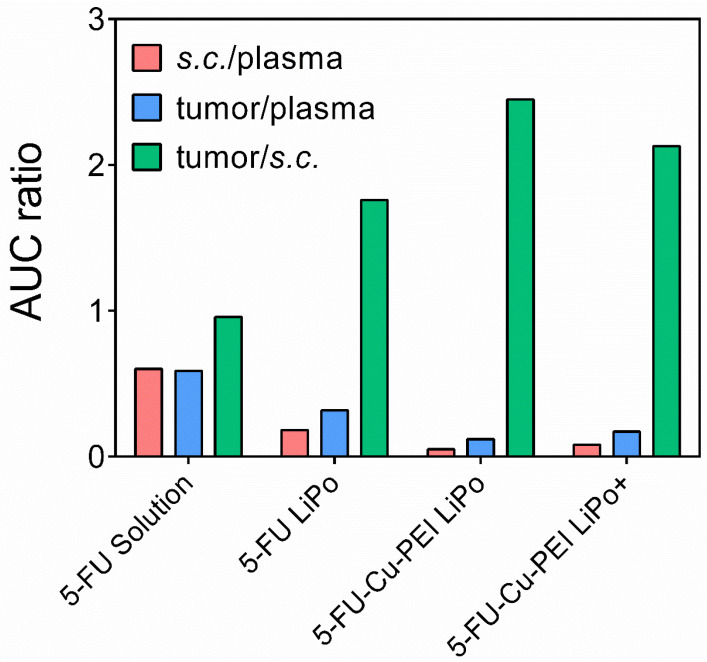
Relative exposure of 5-FU (mean ± SD) among plasma, healthy *s.c.* tissue and tumor following an *i.v.* dose of various 5-FU formulations (10 mg/kg). Data represent mean ± S.D. (*n* ≥ 5).

To clarify whether there was a greater transfer of 5-FU into tumor tissue compared with healthy tissue for liposomal formulations, the relative exposure of 5-FU was calculated as the ratios of AUC_tumor_/AUC_plasma,_ AUC*_s.c._*/AUC_plasma_ and AUC_tumor_/AUC*_s.c._* and is presented in Figure 5. A statistically greater (*p* < 0.05) accumulation into the tumor relative to *s.c.* tissue was observed for liposomal PEI-Cu-5-FU in comparison to the 5-FU solution, as demonstrated by an increase in the ratio of exposure (i.e., AUC_tumor_/AUC*_s.c._*_tissue_) from 0.958 ± 0.244 for the 5-FU solution to 2.45 ± 0.77 for 5-FU-Cu-PEI LiPo and 2.13 ± 0.51 for 5-FU-Cu-PEI LiPo+.

### 3.4. Efficacy of Liposomal 5-FU Formulations

**Figure 6:**
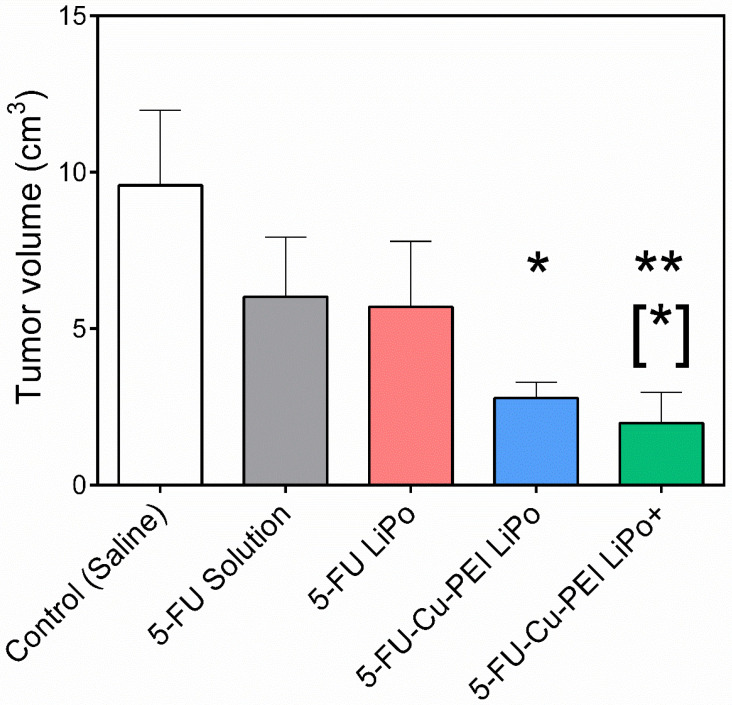
Tumor volume of rats administered 5-FU formulations versus a saline control on day 12, following five consecutive days of dosing. Statistical significance is represented as * *p* < 0.05, ** *p* < 0.01 in contrast to the saline control; [*] *p* < 0.05 in contrast to the 5-FU solution. Data represented as mean ± S.D., *n* = 6.

Tumor growth was measured following five consecutive days of daily dosing of each 5-FU formulation (Figure 6). The unformulated 5-FU solution exerted a regressive effect on tumor growth, with no further enhancement in efficacy observed for 5-FU LiPo. Importantly, complexed 5-FU with Cu-PEI exerted a statistically significant reduction in tumor volume compared to the control group, with 5-FU-Cu-PEI LiPo+ demonstrating optimal efficacy through a statistically significant reduction (*p* < 0.05) in tumor volume compared to the 5-FU solution, with a final tumor volume of 1.98 ± 0.98 cm^3^.

## 4. Discussion

The present study utilized microdialysis to evaluate the uptake and disposition of the anti-cancer drug 5-FU when encapsulated within both neutral and cationic liposomes into tumor tissue. Unlike other tumor-targeting studies that measure the distribution of anti-cancer drugs within tumors by measuring both the free and encapsulated drug, microdialysis affords a unique advantage by quantifying only the free drug that is exposed to the tumor tissue. Thus, in this study, by virtue of the microdialysis technology, the availability and time course of 5-FU in tumor and adjacent healthy *s.c.* tissue were compared after measuring simultaneously the free concentrations of 5-FU in the interstitial fluid of both tissues.

### 4.1. Rationale for Liposomal 5-FU Polymer Complexes

It was hypothesized that 5-FU disposition within tumor tissue would be greater for liposomal formulations owing to the ability for liposomes to prolong retention of the encapsulated drug in plasma and their capacity to preferentially localize within solid tumors due to the enhanced permeability and retention (EPR) effect. Subsequently, liposomes were fabricated with varying lipid compositions and surface charges to investigate this effect. Since 5-FU is a small molecule with a logP of ~0.9 it does not associate with the lipid bilayer [31]; rather is encapsulated within the aqueous liposome core. As such, maximizing the aqueous volume is of major importance for optimizing 5-FU encapsulation efficiency. It is well known that by adding cholesterol, the liposomal membrane becomes less rigid and the internal aqueous volume decreases [32]; so, formulations with a higher level of cholesterol in the lipid phase showed a lower encapsulation efficiency of 5-FU. Liposomes with stealth-like characteristics through modification with polyethylene glycol (PEG) can minimize their capture by macrophages and extend circulation time, thus further enhancing their availability for permeation into and retention by tumor tissue [33]. As such, the PEG content was varied within liposomes, with an optimal concentration of 5 mol%, leading to an encapsulation efficiency of 16.0 ± 2.2% (Figure 1).

In vitro-release studies revealed that more than half of the encapsulated 5-FU was released from neutral liposomes within 40 min. Due to the chemical nature of 5-FU it can freely pass through the liposome membrane and therefore, simply incorporating 5-FU alone into liposomes was considered as an unsatisfactory approach to significantly extend its in vivo half-life, compared to the administration of a 5-FU solution. However, due to the deprotonation of 5-FU in neutral aqueous media, complexes can be readily formed with metal ions. In this study, 5-FU was complexed with ternary copper together with polyethyleneimine (PEI) to create a considerably larger molecular species than 5-FU alone [25], where once entrapped within liposomes, the drug was retained for longer than the pure drug, as highlighted by the in vitro release profiles of the 5-FU-Cu-PEI LiPo and 5-FU-Cu-PEI LiPo+ in Figure 2. Importantly, the rate of 5-FU release from both neutral and cationic liposomes was significantly impeded when formulated with the PEI complex, which suggests that this approach is more viable for prolonging 5-FU exposure to tumor tissue. No changes were observed in the in vitro release kinetics between 5-FU-Cu-PEI from neutral and cationic liposomes since the inability of 5-FU-Cu-PEI to permeate the lipid bilayer was modulated by the size of the polymer complex and not controlled via electrostatic interactions.

### 4.2. Liposomal 5-FU Polymer Complexes Enhance Tumor Targeting

When incorporated into neutral liposomes alone, 5-FU was rapidly cleared in vivo with no significant increase in 5-FU exposure to tumor tissue, compared to the 5-FU solution. The inability for 5-FU LiPo to increase exposure was attributed to the rapid release kinetics, whereby the majority of the drug was released within one hour. As such, liposomes could not facilitate an EPR effect due to the release of the encapsulated cargo prior to exposure to the tumor tissue. However, when 5-FU was incorporated as a complex with Cu-PEI in neutral and cationic liposomes, the AUC of 5-FU in plasma compared with 5-FU solution increased 8.6- and 4.8-times, respectively. This translated into a significant increase in the exposure of 5-FU in tumor tissues compared with 5-FU solution, by ~80% and 50%, respectively (Figure 3 and Table 1). It was found that 5-FU-Cu-PEI LiPo+ (CL, 8.80 ± 1.11 mL/min) was cleared from the plasma faster (CL, 8.80 ± 1.11 mL/min; *p* < 0.05) than 5-FU-Cu-PEI LiPo (CL, 5.24 ± 1.36 mL/min). This may have been due to cationic liposomes interacting nonspecifically with anionic species (e.g., plasma proteins) [34], leading to a more rapid clearance from circulation by the reticuloendothelial system [35,36,37]. Despite this, the MRT of 5-FU in tumor tissue was similar with both liposomal 5-FU polymer complex formulations (99.5 ± 24.8 min vs. 96.4 ± 27.0 min).

An important aspect of the microdialysis technology is the capacity to sample healthy tissue and the ECF surrounding the tumor tissue, to ultimately quantify and compare the drug concentration within both tissues. Liposomal 5-FU polymer complexes significantly increased the AUC ratio between healthy *s.c.* and tumor tissue compared to both the 5-FU solution and 5-FU LiPo. This confirmed the capacity for the liposomes in this study to passively target tumor tissue and enhance the permeation and uptake of 5-FU into tumor tissue. However, it is critical that 5-FU was encapsulated within the liposomes as a polymer complex to prevent premature drug release and ensure the drug was exposed to the tumor tissue.

### 4.3. Cationic Liposomal 5-FU Polymer Complex Improves Anti-Tumor Efficacy

Tumor growth was significantly slowed when 5-FU was encapsulated within liposomal formulations as a polymer complex in response to the increased permeation and retention of the drug within the tumor tissue. Of the two liposomal 5-FU polymer complex formulations, cationic liposomes were the most effective over the dosing period. A tumor volume of 1.98 ± 0.98 cm^3^ was recorded for 5-FU-Cu-PEI LiPo+, compared to 2.78 ± 0.50 cm^3^ for 5-FU-Cu-PEI LiPo, and although not statistically significant at day 12, it appears that the trend would have continued and that the anti-tumor efficacy would have been greater for cationic liposomes if the study had been followed for a longer period of time. This finding was despite the 5-FU concentration within tumor tissue being greater for 5-FU-Cu-PEI LiPo, compared to 5-FU-Cu-PEI LiPo+ (Figure 4).

Cationic liposomes are reported to have the ability to target tumor vasculature [8], due to their positive surface charge, and their selective binding to the net negatively charged surface of angiogenic endothelial cells in the tumors. However, cationic liposomes did not further enhance distribution of 5-FU into tumor interstitial fluid compared to neutral liposomes, as reflected by the lack of any significant differences in the respective values of exposure (AUC) and residence time (Table 1). It may be that 5-FU within the cationic liposomes enters endothelial cells of the tumor vasculature via charge-mediated binding, endocytosis, and endosomal drug release thereby being unavailable for sampling by the microdialysis probes (molecular weight cut-off of 20 kDa). However, once the binding is saturated, cationic liposomes may extravasate into the interstitial fluid of the tumor tissue and release 5-FU within. This is supported by the increased exposure of 5-FU (sampled by microdialysis, which only dialyses released 5-FU) in tumor tissue from cationic liposomes (189 ± 25 µg.min/mL) compared with that from the 5-FU solution (127 ± 30 min*µg/mL). This is in agreement with Lila et al. [15], who suggested that cationic liposomes can realize the dual targeting to both tumor vasculature and the tumor cells themselves, the latter via the interstitium of the tumor. Therefore, 5-FU-Cu-PEI LiPo+, while not inducing a longer residence time in plasma compared to 5-FU-Cu-PEI LiPo, may have a greater overall exposure within the tumor, both to the tumor cells themselves (as an EPR effect that depends on extravasation), and to the endothelial cells of the tumor vasculature due to the electrostatic interaction. However, the proposed dual targeting of cationic liposomes to both tumor vasculature and tumor cells could be further confirmed by experiments such as visualization of fluorescently-labeled neutral or cationic liposomes in the tumor using an in vivo imaging system, or by injecting fluorescent neutral or cationic liposomes into tumor-bearing mice, and using fluorescence with confocal microscopy to compare the accumulation of the liposomes in the tumor vasculature.

### 4.4. Limitations and Future Directions

A key limitation of microdialysis is that the probe takes samples from a specific part of the tumor tissue, and therefore the concentrations obtained may not reflect the overall distribution of drug throughout a tumor. The distribution of liposomes/anticancer drug into different parts of a tumor can vary; for example, some parts of the tumor may have a greater blood supply which may subsequently affect the extent of drug distribution. As such, future studies should utilize microdialysis to probe various regions within the tumor to quantify liposome and drug distribution throughout the tumor tissue. Doing so will elucidate whether specific formulation approaches provide enhanced drug exposure to the entire tumor tissue rather than just localized regions. Nevertheless, upon completion of the experiments the tumors were removed and sliced for visual inspection and appeared homogeneous. In contrast, visualization using fluorescently-labelled liposomes should be considered in future as a qualitative approach to investigating the distribution of liposomal formulation throughout the entire tumor. Despite this, microdialysis offers the advantage of gaining dynamic, time-dependent data of unbound drug concentrations and provides a key potential for clinical studies in humans, that is not realistic for fluorescently-labelled delivery systems. Furthermore, conventional fluorescent experimental approaches for assessing distribution typically only afford one or a few time points in order to avoid excess animal wastage and are unable to quantify the concentration of unbound drug within the tumor tissues [37].

The current study highlighted the potential for liposomal 5-FU polymer complexes to increase tumor-targeting and subsequent anti-tumor efficacy. However, further work is required to validate the efficacy and safety of this approach in treating solid tumors. Future attention will be placed on assessing time-dependent efficacy in reducing tumor growth over a prolonged study period, monitoring survival, and quantifying toxicity through changes in body weight and key hematological biomarkers, as well as observing drug-induced damage to intestinal mucosa. These future studies will allow correlations to be drawn between drug disposition within the tumor and efficacy versus toxicity, which will ultimately drive the development of liposomal 5-FU polymer complexes towards translation to the clinic.

## 5. Conclusions

Liposomes were optimized for loading anticancer drug, 5-Fu and found to prolong the plasma and tissue exposure of 5-Fu. Complexation of 5-Fu with Cu-PEI prior to incorporation into neutral and cationic liposomes resulted in prolonged systemic circulation and accumulation in a tumor microenvironment compared to 5-Fu alone and in liposomes. Microdialysis was successful in measuring released 5-Fu in the tumor microenvironment as a function of time, allowing pharmacokinetic parameters to be determined and compared to those of healthy tissue and to plasma exposure. Both neutral and cationic liposomes were found to improve tumor exposure to 5-Fu compared to healthy tissue and resulted in statistically reduced tumor growth compared to 5-Fu solution. Thus, liposomal 5-Fu polymer complexes demonstrate potential of nanomedicine for improved delivery of anticancer drugs.

## Figures and Tables

**Table 1:** Comparison of pharmacokinetic parameters for 5-FU with respect to concentrations in plasma and extracellular fluid from healthy *s.c.* tissue and tumor tissue following an i.v. dose of various 5-FU formulations. Data represent mean ± S.D. (*n* = 6).

Pharmacokinetic Parameter	Solution	5-FU LiPo	5-FU-Cu-PEI LiPo	5-FU-Cu-PEI LiPo+
**AUC_0–∞_ (µg.min/mL)**				
Plasma	238 ± 70	530 ± 93 **	2040 ± 620 ** (*)	1150 ± 165 *** (**)
*s.c.*	137 ± 39	91.4 ± 16.0	99.2 ± 37.0	93.4 ± 29.1
Tumor	127 ± 30	159 ± 49	230 ± 74 **	189 ± 25 *
**Half-life (min)**				
Plasma	13.4 ± 6.2	19.6 ± 4.2	31.8 ± 3.9** (*)	29.2 ± 9.8 **
*s.c.*	15.3 ± 3.2	16.0 ± 2.0	53.1 ± 2.7*** (***)	32.9 ± 19.7
Tumor	21.2 ± 3.0	29.0 ± 11.0	54.2 ± 9.3** (*)	59.9 ± 19.4 * [*]
**MRT (min)**				
Plasma	15.1 ± 1.3	19.3 ± 2.7	31.1 ± 5.4 ** (*)	13.2 ± 1.5 (**) [*]
*s.c.*	26.3 ± 9.2	30.1 ± 4.0	69.9 ± 8.5 *** (**)	39.3 ± 14.9 [*]
Tumor	36.1 ± 10	56.1 ± 12.3	99.5 ± 24.8 *** (**)	96.4 ± 27.0 ** (*)
**C_max_ (µg/mL)**				
*s.c.*	5.19	3.17	2.46	5.02
Tumor	3.20	3.05	2.03	3.45

Note: * *p* < 0.05; ** *p* < 0.01; *** *p* < 0.001, neutral liposomal 5-FU, neutral or cationic liposomal PEI-Cu-5-FU compared against a solution of 5-FU; (*) *p* < 0.05; (**) *p* < 0.01; (***) *p* < 0.001, neutral or cationic liposomal PEI-Cu-5-FU compared against neutral liposomal 5-FU; [*] *p* < 0.05, cationic liposomal PEI-Cu-5-FU compared against neutral liposomal PEI-Cu-5-FU; AUC_0-∞,_ area under the curve; MRT, mean residence time.

## Data Availability

Not applicable.
